# Ceiling effects in the Movement Assessment Battery for Children-2 (MABC-2) suggest that non-parametric scoring methods are required

**DOI:** 10.1371/journal.pone.0198426

**Published:** 2018-06-01

**Authors:** Blandine French, Nicole J. Sycamore, Hannah L. McGlashan, Caroline C. V. Blanchard, Nicholas P. Holmes

**Affiliations:** School of Psychology, University of Nottingham, Nottingham, United Kingdom; University of Illinois at Urbana-Champaign, UNITED STATES

## Abstract

Initially designed to identify children’s movement impairments in clinical settings, the Movement Assessment Battery for Children-2 (MABC-2) is also widely used to evaluate children’s movement in research. Standardised scores on the test are calculated using parametric methods under the assumption of normally-distributed data. In a pilot study with thirty five 8–10 year old children (i.e., in Age Band 2 of the MABC-2), we found that maximal performance was often reached. These ‘ceiling effects’ created distributions of scores that may violate parametric assumptions. Tests of normality, skew, and goodness-of-fit revealed this violation, most clearly on three of the eight sub-tests. A strong deviation from normality was again observed in a sample of 161 children (8–10 years, Experiment 1), however ceiling effects were reduced by modifying the scoring methods, and administering items designed for older children when maximal performance was reached. Experiment 2 (n = 81, 7–10 years) further refined the administration and scoring methods, and again improved the distributions of scores. Despite reducing ceiling effects, scores remained non-parametrically distributed, justifying non-parametric analytic approaches. By randomly and repeatedly resampling from the raw data, we generated non-parametric reference distributions for assigning percentiles to each child’s performance, and compared the results with the standardised scores. Distributions of scores obtained with both parametric and non-parametric methods were skewed, and the methods resulted in different rankings of the same data. Overall, we demonstrate that some MABC-2 item scores are not normally-distributed, and violate parametric assumptions. Changes in administering and scoring may partially address these issues. We propose that resampling or other non-parametric methods are required to create new reference distributions to which an individual child’s performance can be referred. The modifications we propose are preliminary, but the implication is that a new standardisation is required to deal with the non-parametric data acquired with the MABC-2 performance test.

## Introduction

The need for valid and reliable tests is essential for professionals measuring the motor abilities of developing children. Many instruments have been developed to assess movement in children, in the form of questionnaires (e.g., the Checklist of the Movement Assessment Battery for Children, 2^nd^ Edition, MABC-2 [1]; Teacher Estimation of Activity Form, TEAF, [2]), or full movement assessments (e.g., the Test of Gross Motor Development, TGMD, [3]; the MABC-2 performance test, [1]). The MABC-2 measures movement in children 3–16 years of age, and is the most widely used instrument to identify movement impairment by clinicians and researchers in children with developmental coordination disorder (DCD) and other suspected developmental disorders [4]. As yet, no test or checklist meets the psychometric requirements for population screening for DCD [5]. While the reliability of the first edition of the MABC was tested and reported in the MABC-2 Manual [1], little research has yet been conducted regarding the psychometric properties of the MABC-2 [6]. The present work assesses one of the assumptions underlying the analysis of data arising from the MABC-2, namely the parametric distribution of scores arising from the performance tests.

In the MABC-2 performance test (Table 1), impairments are measured in three movement domains ('components'): manual dexterity (MD), aiming and catching (AC), and balance (B). These components include two (AC) or three (MD, B) different tasks ('items'), and the scoring of each item results in one or more raw performance scores. By referring to standardised tables, these raw scores are converted into an integer item standard scores (ISS) with a mean of 10 and standard deviation of 3. This is done under the parametric assumptions of a normal distribution, separately for the different items, age bands, and for individual age groups within each band. Some of the item standard scores are averaged across the two hands or feet, and if this average is not an integer, the averaged item standard score is *rounded away from 10*. Item standard scores (ISS) for each item in each component are summed to produce component standard scores (CSS). Finally, standard scores across the three components are added together, in spite of the aiming and catching component comprising only two items, to produce the overall total standard score (TS). While the MABC-2 manual states that the standardisation data were transformed to create normal distributions, no further detail about this process is available.

**Table 1:** Test items of the MABC-2 for Age Band 2 and 3.

Section	Age Band 2 (7–10 years)	Age Band 3 (11–16 years)
Manual Dexterity (MD)	1: Placing pegs on a board *two trials per hand- best score*	
	2: Threading lace *two trials- best score*	
	3: Drawing trail (level 2) *two trials- best score*, *no second trial if performed at best*	3: Drawing trail (level 3) *two trials- best score*, *no second trial if performed at best*
Aiming and Catching (A&C)	1: Catching with two hands	
	2: Throwing bean bag onto mat	
Balance (B) *For all tasks*: *two trials—best score*, *no second trial if performed at best*	1: One-board balance *two trials per leg*	
	2: Walking heel to toe forward	2: Walking heel to toe backward
	3: Hopping on mats *two trials per leg*	3: Zig-zag hopping on mats *Two trials per leg*

The present report focuses on Age Band 2 items, since we have tested over three hundred children aged 7–10 years on these items as part of a larger, ongoing project. Six out of the eight items in this Age Band of the MABC-2 require two trials, and only the best-performed trial contributes to the final score (see Table 1 for a summary of the items). For four of these items—drawing a trail (MD3), one board balance (B1), walking heel-to-toe forwards (B2), and hopping on mats (B3)—when a child performs perfectly on the first trial, the examiner is instructed not to administer a second trial. For three of the items (MD1, B1, B3), the child first performs two trials of the item with their dominant (or preferred) hand or foot, then performs two further trials with their non-dominant (or non-preferred) hand or foot. For the balance items, each of the two feet are used, with the child choosing which to start on (i.e., the preferred foot). Regardless of this preference, the data are later re-coded into the best-performing and the other foot.

Despite the apparent complexity of the administration and scoring system, it is in fact straightforward to implement, data recording is by pen-and-paper, and the method has been fully standardised. Nevertheless, the approach may lead to mis-estimation of children’s abilities because of ‘ceiling effects’. Ceiling effects occur when the scores of a relatively large proportion of a sample are in the upper range of the measurement scale. When a dependent variable is no longer measurable or estimable, due to limitations in the instrument’s sensitivity, or the number of observations taken at the upper range, variance will decrease, and the distribution will compress at the upper range, becoming skewed. The highest scores on measures with ceiling effects are therefore unable to assess participants’ true ability (see, e.g., Holmes [7], for further discussion on ceiling effects).

We believe that the MABC-2 scoring system is prone to ceiling effects for four reasons. First, luck is a factor in all human performance. Should a child get ‘lucky’ on their first trial of a given item, reaching the maximum possible performance, it is not advisable to stop data collection, since this child’s score would then always be recorded as maximal, with no variance, and no estimate of the role of luck. This ‘stopping rule’ likely creates ceiling effects. Second, taking forward only the best performance of two validly-performed trials for some items also likely leads to ceiling effects. Third, children who perform the best will progress through the items more rapidly, performing fewer trials, on average, than children who perform worse. The worst-performing children will likely take longer, have to perform more trials, and may as a consequence become more tired and less motivated, further impacting performance, while the best will perform fewer trials overall. Fourth, the test items may be too easy, especially for the oldest children in a given Age Band.

On the whole, then, the MABC-2 seems designed to discriminate between performances only at the lower end of the scale, while inflating towards ceiling performances at the upper end. While this is the, perfectly valid, aim of the MABC-2, in calculating the total score the raw data are processed and selected. It is perhaps unlikely, therfore, that the resulting distributions are normal. The potential biasing effects of using normal distributions to interpret MABC-2 scores is not known.

Our study investigates the distributions of scores in Age Band 2 of the MABC-2. First, we analysed a smaller dataset collected using the standard MABC-2 protocols (Pilot Experiment) in order to assess any ceiling effects or non-normal distributions. Second, in Experiment 1, we investigated several alternative methods of normalising the distributions by either changing the test items themselves to assess a wider range of movement performance (Experiment 1), or by changing the way the items are scored (Experiment 2). Third, we investigated an entirely novel means of calculating standardised scores, using a non-parametric resampling approach. For all analyses, we used the MABC-2 to quantify the movement coordination of a total of 277 children across a wide range of movement performance, including 12 children with a formal diagnosis of DCD or dyspraxia. An overview of the study is provided in Table 2. For detailed description and instructions for performing the individual movement tests, please see the MABC-2 manual [1].

**Table 2:** An overview of the study samples, tasks, and methods.

Experiment	Sample			MABC-2 tasks		Notes
	N	Age (years)	Gender	Age Band 2	Age Band 3	
Pilot	35	8–10	20F, 15M	MD1-3, A&C1-2, B1-3	-	Standard instructions
Experiment 1	161	8–10	103F, 58M	MD1-3, A&C1-2, B1-3	MD3, B2, B3	Standard instructions for Age Band 2 Additional trial at Age Band 3
Experiment 2	81	7–10	44F, 37M	MD3, B2, B3	MD3, B2, B3	2 trials of Age Band 2 1 trial of Age Band 3 15 steps on task B2
Re-analysis	227	7–10	134F, 93M	MD1-3, A&C1-2, B1-3	-	Standard analysis compared with non-parametric analysis

## Pilot experiment: Standard MABC-2 protocols

### Methods

#### Participants

35 children took part in our pilot study (mean±SD age = 9.5±0.5 years, 20 females). The participants were recruited from a primary school in the UK. Age was the only exclusion criterion, with the study focusing on 8–10 year olds.

In line with DSM-5 criteria for identifying DCD [8], social economic status (using Income Deprivation Affecting Children Index (IDACI), 2015), measures of ADHD tendencies and intellectual abilities were taken in all participants. This study was approved by the research ethics committee at the University of Reading (2013-148-NH).

#### Apparatus

**MABC-2 performance test**. The MABC-2 is designed to identify impairments in motor performance of children from 3 to 16 years of age and is divided into three age bands (age band 1: 3–6 years, age band 2: 7–10 years, age band 3: 11–16 years). The tasks are different for each age band; for the purpose of this study, only materials for age band 2 and 3 tasks of age band 3 were used. While the MABC-2 is composed of a performance test and a checklist, only the performance test was used in the current study, which we refer to as MABC-2. The 8 tasks of the performance test assess three domains of fine and gross motor skills. For age band 2, these tasks are as follows—manual dexterity: placing pegs (MD1), threading lace (MD2), drawing a trail (MD3); aiming and catching: catching with two hands (A&C1) and throwing a bean bag onto a mat (A&C2) and balance: one-board balance (B1), walking heel to toe forward (B2), and hopping on mats (B3). In order to score performance, each task provides one or more raw scores which are processed and converted into an item standard score with a mean of 10 and standard deviation of 3. The item standard scores are then summed for each task in the three domains (the component score), converted again into 3 standard scores (mean = 10, SD = 3), and finally linked to percentiles, under the assumption that the data are normally distributed. Total test score is similarly calculated by adding up the three domain scores. Scores at or below the 5^th^ percentile are often used to indicate children with severe movement difficulties, and scores between the 6^th^ and the 16^th^ percentiles inclusive are considered to have borderline movement difficulties (the often-cited standard cut off value of 15% is not available in the MABC-2, [8]). To minimise experimenter error, we have created macros in Microsoft Excel to automate the calculation of MABC-2 scores and percentiles [9].

#### Design & procedure

After obtaining written, informed consent from all parents, and written assent from children, the children were tested in a primary school. Children were individually assessed on the MABC-2 following the test manual instructions. For all our experiments, the examiners were experienced in the administration of standardised tests with children, including the MABC-2. The children also completed a reaching and grasping task and the BAS-2. Conners 3 AI questionnaires were completed by teachers.

#### Analysis

In order to assess the distribution of the data, we took three measurements. First, skewness of the distribution measures its asymmetry, in which a skew of 0 shows a perfectly symmetrical distribution and a skew greater than 1 shows a strong asymmetrical distribution. Second, a statistical test of normality was performed—the Shapiro-Wilk for small samples (n≤50) or Kolmogorov-Smirnov for large samples (n>50), in which significant p-values indicate departure from a normal distribution. Third, a Chi-square goodness-of-fit test was used to compare the distribution of real data with a data set simulated in MATLAB (MathWorks, Natick, USA), which was generated from a normal distribution with the same N, mean, and SD as the real data, sampled at discrete (i.e., plausible) values for each raw score. A significant Chi-square goodness-of-fit test indicates that the real data do not fit the data simulated from this normal distribution.

### Results & discussion

**Fig 1:**
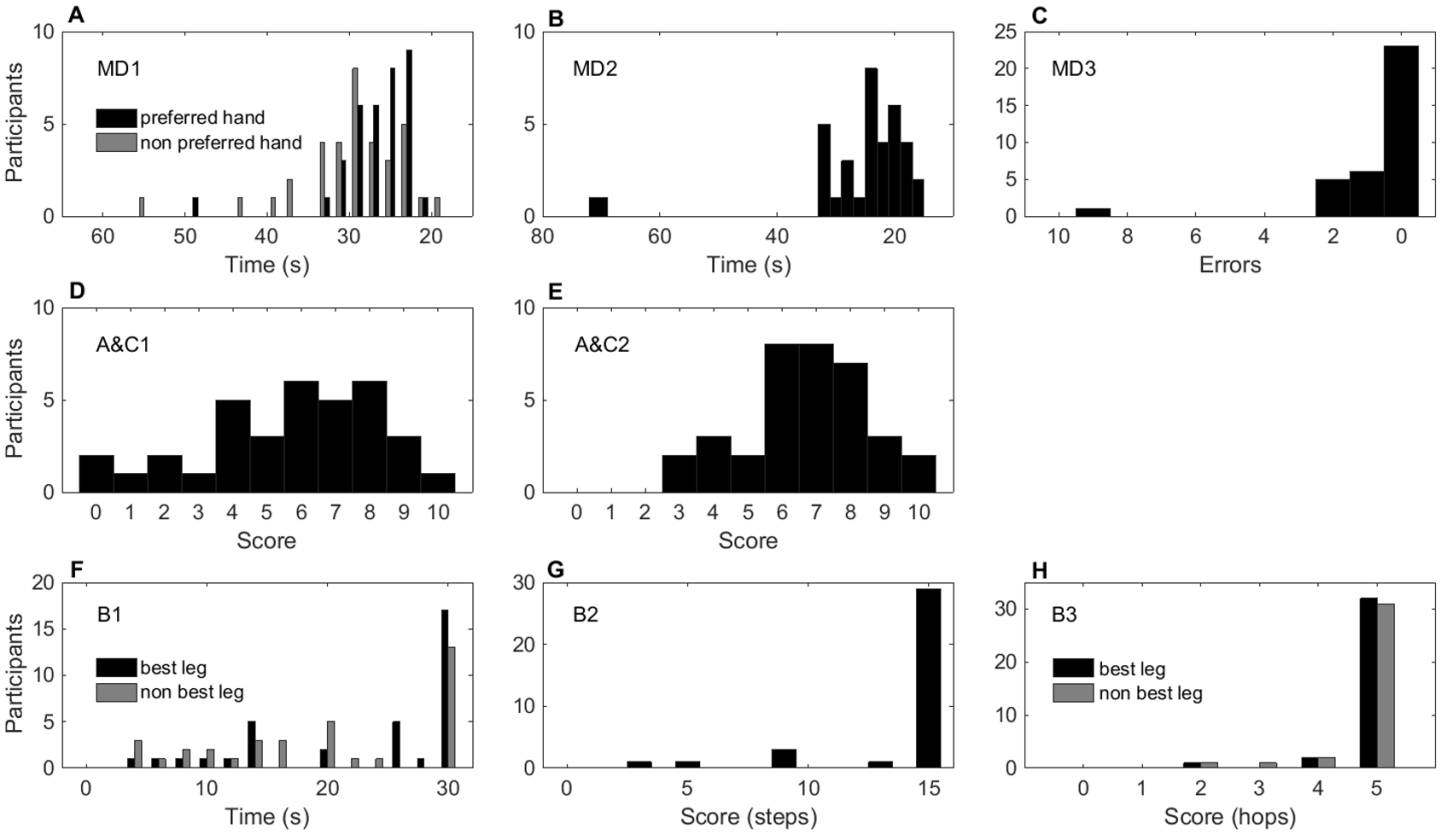
Distribution of the scores of the eight MABC-2 items, pilot study (n = 35). The y-axis shows the number of participants achieving a given raw score (x-axis). Panels A-C, manual dexterity; D-E, aiming and catching; F-H, balance items. Good performance is represented on the right, and poor performance on the left of each x-axis. Ceiling effects are seen in a greater proportion of the distribution on the right of each histogram (e.g., panel G). Items where both hands (or legs) were scored are shown in the same panel, with the preferred or best limb in black and the non-preferred or other limb in grey.

Our pilot study (n = 35) tested 8–10 year old (age band 2) children on the MABC-2. We observed that a high percentage of children reached the top scores on 4 tasks (MD3 66%, B1 48% best leg and 37% other leg, B2 83%, B3 91% best leg and 88% other leg, Fig 1, panels C, F, G, and H, and Table 3). For each of these tasks, a strong skew (over 1), a significant departure from normality (Shapiro-Wilk), and a significant Chi-square goodness-of-fit test demonstrated that data from those tasks were not normally distributed (Table 3). Given the small sample size of our pilot study, these observations needed to be confirmed and refined in a larger sample.

**Table 3:** Skewness, test of normality and chi square results of MABC-2 tasks for the pilot study.

	MD1		MD2	MD3	A&C1	A&C2	B1		B2	B3	
	Pref.	Non pref.					Best leg	Other leg		Best leg	Other leg
**Pilot**											
**Skew (SE)**	2.81 (0.40)	1.78 (0.40)	3.65 (0.40)	4.20 (0.40)	-0.60 (0.40)	-0.29 (0.40)	-1.00 (0.40)	-0.30 (0.40)	-2.57 (0.40)	-4.59 (0.40)	-3.52 (0.40)
**Shapiro-Wilk** Df = 35 (p)	.743 (.001)	.867 (.001)	.645 (.001)	.468 (.001)	.946 (.085)	.955 (.162)	.756 (.001)	.870 (.001)	.464 (.001)	.289 (.001)	.366 (.001)
**Kolmogorov-Smirnov** Df = 35 (p)	.172 (.011)	.187, (.035)	.210 (.001)	.330 (.001)	.148 (.50)	.145 (.60)	.285 (.001)	.230 (.001)	.484 (.001)	.517 (.001)	.510 (.001)
**X**^**2**^ (Df) (p)	6(4), (.155)	4(5), (.464)	8(5), (.149)	23(2), (.05)	2(4), (.73)	0.45(4), (.97)	174(5), (.001)	157(5) (.001)	107(3), (.001)	5(1), (.035)	7(1), (.007)
**% at ceiling**	N/A	N/A	N/A	66	2.8	5.7	48	37	83	91	88

MD: Manual dexterity; A&C: Aiming and catching; B: Balance; Pref.: Preferred; Df: degrees of freedom. N/A: Not applicable—no fixed ceiling.

We observed in the pilot experiment that the best performance was often reached on the first trial of an item and, across the two trials, a high percentage of children performed at ceiling (especially on the MD3, B1, B2, and B3 items). Ceiling effects have previously been observed in studies using the first version of the MABC. Miyahara and colleagues [10] reported that all 8 and 9 years old (n = 45) performed B2 and B3 items without error. Other studies have also found a substantial ceiling effect on B2 [11], observing up to 92% of children performing at ceiling [12]. Following reviews addressing the validity and reliability of the test, the second edition of the MABC was published. The main differences were a reduction in the number of Age Bands (from four to three), the revision and addition of new items, inclusion of a more representative standardization sample, and the rearrangement of the sub-tests [6]. The need to further adjust some of these items due to ceiling effects was also suggested and addressed [6,11]. However, our pilot experiment results suggest that the problems raised in the first edition remain in the second.

## Experiment 1: Testing children on higher Age Band items

According to the MABC-2 manual, when a child has failed on the first trial and is reluctant to proceed, three sorts of adaptation are recommended to gain more information about the child’s movement skills. These are:

1. Testing the child at a lower Age Band of the test (if available)
2. Modifying or adapting the test items
3. Providing the child with assistance or feedback during performance

As suggested in the first adaptation, testing children at a lower Age Band is recommended when the child is unable to complete the item at her or his age level. However, is it not clear how data arising from these modifications are to be interpreted. Furthermore, no suggestion is given for children who perform at the maximum level. Following a recommendation from Smits-Engelsman [13], our Experiment 1 (n = 161) investigated the administration of Age Band 3 items when a child performed at best on the first trial. For items which show ceiling effects, testing the children again on a higher Age Band item will allow some refinement in line with valid use of the test. Due to timing constraints and the slightly smaller ceiling effect observed (under 50% of children performed at ceiling), the B1 item was tested as normal in this part of the study, while items MD3, B2, and B3 were tested with an additional trial at the Age Band 3 level when children performed at ceiling on the first trial.

### Methods

#### Participants

161 children (mean±SD age = 9.1±0.7 years, 103 female, 58 male) were recruited from local primary schools (n = 103) and a university database (n = 58). Age was the only exclusion criterion, with the study focusing on 8-10-year olds as part of a larger project interested specifically in this age range. In line with DSM-5 criteria for identifying DCD [8], social economic status (using Income Deprivation Affecting Children Index—IDACI), measures of ADHD tendencies and intellectual abilities were taken for all participants. After obtaining written, informed consent from all parents, written assent was obtained from children. This study was approved by the ethical committee at the University of Nottingham (SoPEC 680). The sample size for the present study was not determined by an *a priori* power analysis, but reflects the total sample available from this larger study. Other datasets from this project have been or will be reported elsewhere, for example [9].

#### Design & procedure

Children were tested in their primary school or within a university laboratory. Children were individually assessed on the MABC-2 following the standard instructions. The instructions were adapted so that a higher Age Band item (Age Band 3) was administered when children performed at ceiling on the first trial of three items (MD3, B2, and B3). The children also completed a reaching and grasping task [9]; the BAS-2 and Conners 3 AI questionnaires were completed by parents (for the university sample) or teachers (the school sample).

### Results & discussion

**Fig 2:**
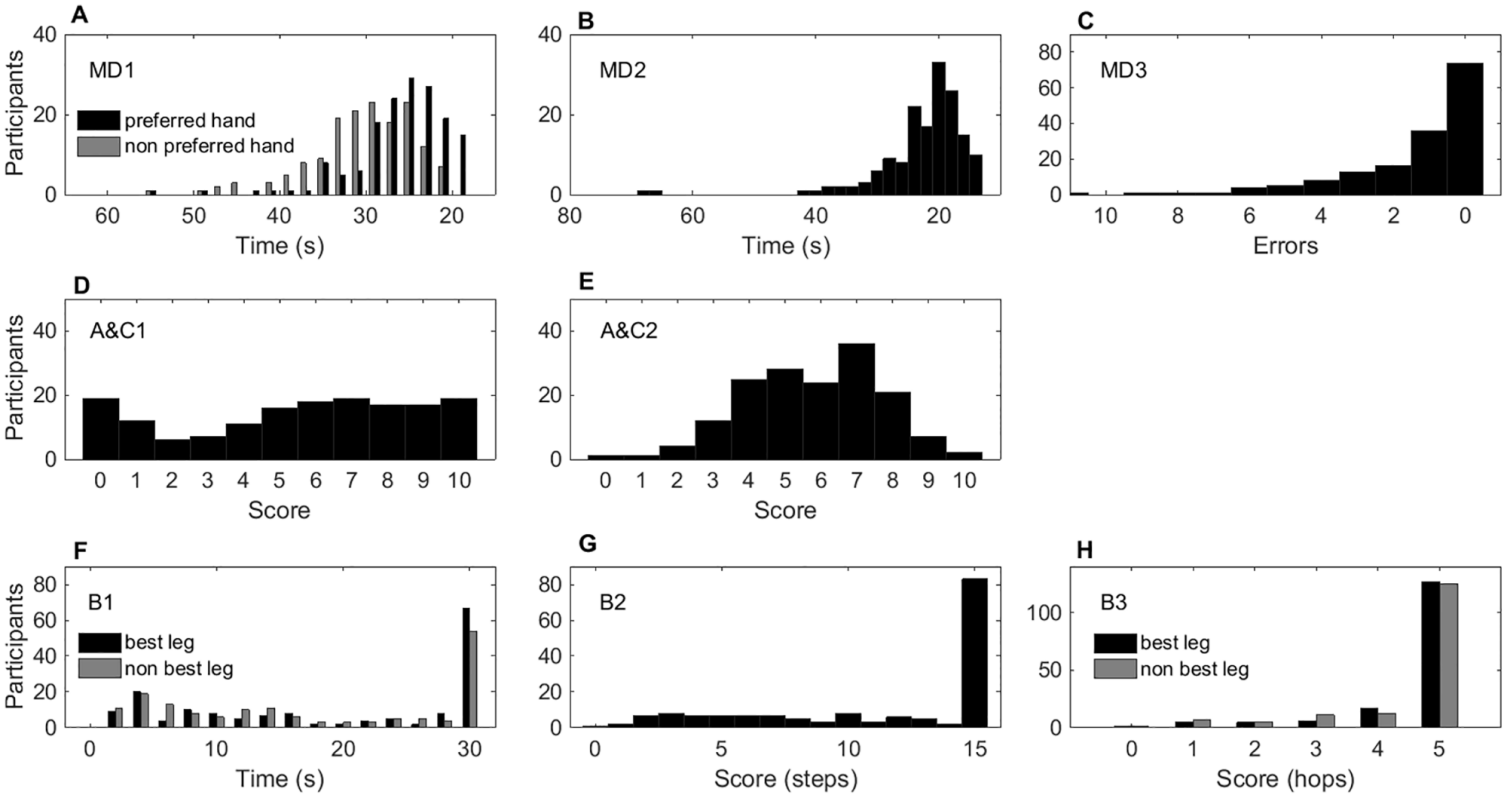
Distribution of the scores of the eight MABC-2 items, Experiment 1 (n = 161). The y-axis shows the number of participants achieving a given raw score (x-axis). Panels A-C, manual dexterity; D-E, aiming and catching; F-H, balance items. Good performance is represented on the right, and poor performance on the left of each x-axis. Ceiling effects are seen in a greater proportion of the distribution on the right of each histogram (e.g., panel G). Items where both hands (or legs) were scored are shown in the same panel, with the preferred or best limb in black and the non-preferred or other limb in grey.

Compared to the pilot experiment data we noticed that, while the ceiling effects were slightly reduced, a high percentage of children still reached the top scores (45% on MD3, 51% on B2, 78% on B3 for their best leg, and 77% on B3 for their other leg; Fig 2, panels C, G, and H, and Table 4). Strong skew (>1), a significant departure from normality (Kolmogorov-Smirnov), and significant Chi-square goodness-of-fit tests demonstrated that data from those three items were not normally distributed in this larger sample (Table 4).

**Table 4:** Skewness, test of normality and chi square results of MABC-2 tasks for the pilot study and Experiment 1.

	MD1		MD2	MD3	A&C1	A&C2	B1		B2	B3	
	Pref.	Non pref.					Best leg	Other leg		Best leg	Other leg
**Exp 1**											
**Skew (SE)**											
Bes**t**	2.19 (0.19)	3.09 (0.20)	3.03 (0.19)	2.24 (0.19)	-.359 (0.19)	-.251 (0.19)	-.361 (0.19)	-.068 (0.19)	-.758 0.19)	-2.65 (0.19)	-2.26 (0.19)
Average	2.12 (0.19)	2.81 (0.20)	3.63 (0.19)	1.91 (0.19)	-	-	.179 (0.19)	.476 (0.19)	-.384 (0.19)	-1.95 (0.19)	-1.74 (0.19)
Band 3	-	-	-	.846 (0.38)	-	-	-	-	.697 (0.29)	-1.91 (0.30)	-2.03 (0.30)
**Kolmogorov-Smirnov** Df = (154–161)											
Best (p)	.139 (.001)	.144 (.001)	.164 (.001)	.268 (.001)	.122 (.001)	.150 (.001)	.253 (.001)	.206 (.001)	.307 (.001)	.454 (.001)	.454 (.001)
Average (p)	.140 (.001)	.136 (.001)	.166 (.001)	.199 (.001)	-	-	.175 (.001)	.142 (.001)	.278 (.001)	.290 (.001)	.308 (.001)
Band 3 (p)	-	-	-	.163(39)(.015)	-	-	-	-	.193(71)(.001)	.35(66)(.001)	.342(66) (.001)
**X**^**2**^ Df = (4–10) Best (p)	7 (.10)	0.88 (.92)	27 (.05)	95 (.001)	51 (.001)	7 (.517)	177 (.001)	137 (.001)	491 (.001)	318 (.001)	251 (.001)
Average (p)	10 (.05)	15 (.05)	36 (.001)	100 (.001)	-	-	91 (.001)	78 (.001)	473 (.001)	164 (.001)	215 (.001)
Band 3 (p)	-	-	-	11, (.05)	-	-	-	-	36 (.001)	34 (.001)	27 (.001)
**% at ceiling**	N/A	N/A	N/A	45	11	1.2	41	33	51	78	77

Experiment 1 includes best of two trials, average across two trials and band 3 trial scores. (.001) indicates p≤.001. N/A: Not applicable—no fixed ceiling.

**Fig 3:**
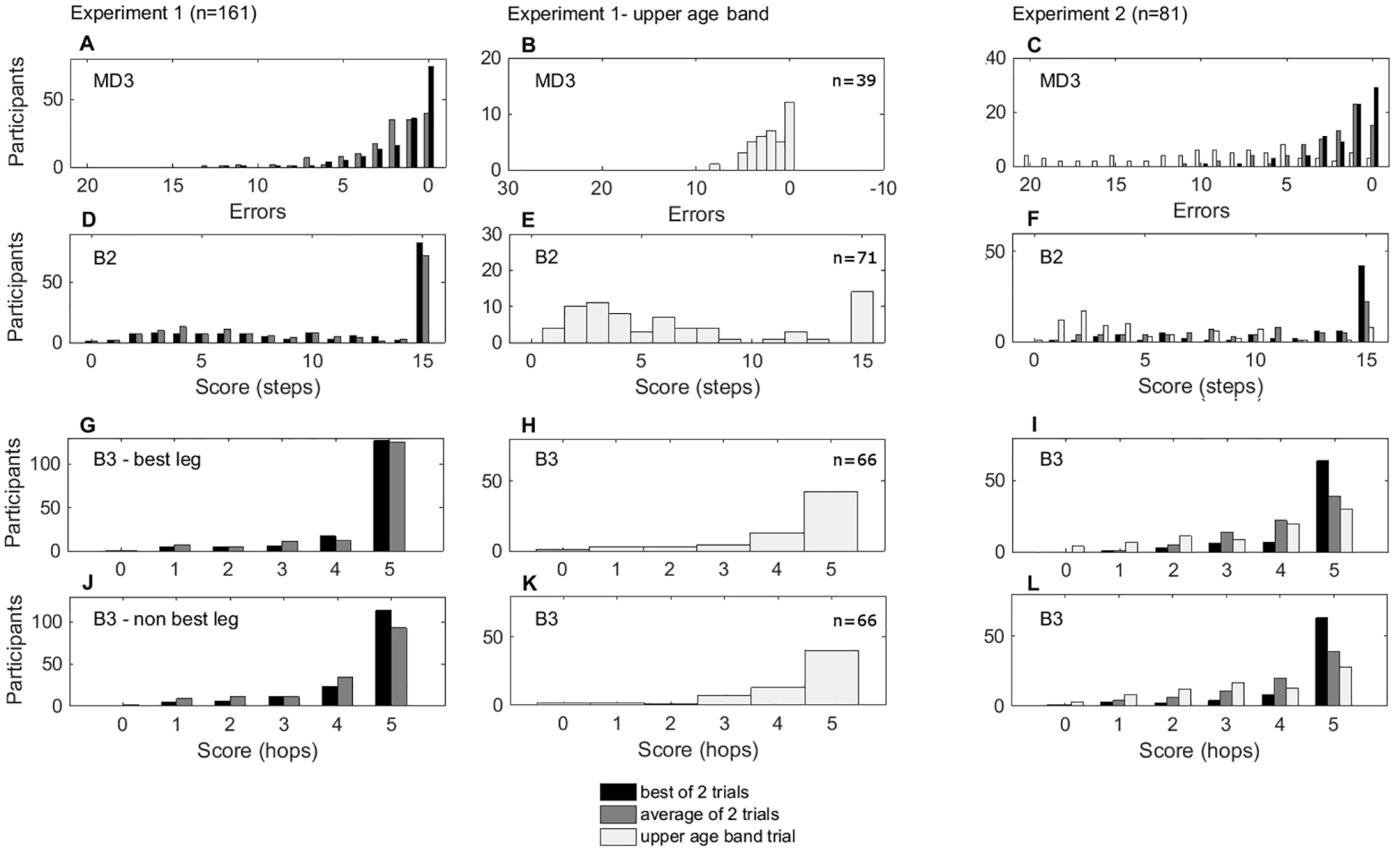
Distribution of the scores of the three MABC-2 items, Experiments 1 (n = 161) and 2 (n = 81) for best of two trials (black), average across trials (mid-grey) and Age Band 3 trial (light grey). The y-axis shows the number of participants achieving a given raw score (x-axis). Good performance is reported on the right and poor performance on the left of each x-axis. Ceiling effects are seen in a greater proportion of the distribution on the right of each histogram (e.g., panel C). Items where both legs were scored are shown in the same panel, with the preferred or best limb in black and the non-preferred or other limb in grey. Three scoring methods are reported for each experiment. Age Band 3 data are reported in separate panels for Experiment 1 due to differences in number of participants.

We performed two further tests to explore alternative methods that could improve the skewed distributions. First, we investigated scoring items taken from the Age Band 3 (11–16 years) when children performed at best on the first trial, and used the second trial (Age Band 3) scores for calculation. This method reduced all the statistical test scores, showing reductions in ceiling effects when Age Band 3 was used (Fig 3 panels A, D, G, H, and Table 4). Secondly, when the children performed two trials on Age Band 2 (for children scoring at best on the first trial, only their first trial score was used), instead of taking just the best score out of the two trials, we took the average of both trials. While this method resulted in data that still departed significantly from a normal distribution, the critical test statistics were again reduced (skew, Kolmogorov-Smirnov, and Chi-square goodness-of-fit), showing a further, independent improvement in normality for the average of the two trials compared to the best of the two (Fig 3 panels B, E, H, K, and Table 4).

## Experiment 2: Modifying the administration and measurement of three MABC-2 items

Experiment 1 demonstrated improvements in the distribution of data by testing children on a more difficult item from Age Band 3, following Smits-Engelsman’s [12] recommendation. Experiment 2 explored modified administration and scoring on the same three MABC-2 items that might further normalize the distribution of their scores. We implemented four main changes:

1. All children received two trials for each item. Perfect performance on the first trial did not lead to cancellation of the second trial. All children also performed one trial on the Age Band 3 item. These changes were made to address the problem of some children doing one trial and some two, ensuring that all children did all trials.
2. Two items were timed (MD3 and B2), allowing both speed and accuracy of performance to be recorded, as this might provide continuous, normally-distributed data. No changes were made to the instructions, and neither timing nor speed were emphasized to the participants.
3. The number of mistakes made were recorded for items B2 and B3, specifically noting down the occurrence (steps) at which errors were made. As opposed to the MABC-2 instructions, an error in B2 did not lead to cancellation of the remainder of the trial.
4. Two experimenters rated both speed and accuracy of performance independently for each child to test for inter-rater reliability.

### Methods

#### Participants

81 children (44 females; mean±SD age = 8.6±1.7 years) were recruited from a University of Nottingham outreach event (Summer Scientist Week). The sample was extended to include 7-year-old children, both for convenience, and to represent the whole age range of the MABC-2 Age Band 2. This study was approved by the research ethics committee at the University of Nottingham (SoPEC 813).

#### Apparatus, materials, and procedure

Three items of the MABC-2 (MD3, B2, and B3) were administered at Age Band 2 and Age Band 3 levels. After obtaining informed consent from all parents, children were individually assessed by experienced examiners. In contrast with the standard MABC-2 instructions, all children performed three trials—two Age Band 2 trials (regardless of performance on the first trial) and one Age Band 3 trial. On B2, children were also asked to perform the entire item (15 steps), regardless of whether or when they made one or more mistakes.

### Results & discussion

Despite this experiment including younger children (therefore including all ages of Age Band 2 of the MABC-2), strong ceiling effects were still observed on the three items when the standard scoring procedure was used (32% in MD3, 50% in B2, 79% in B3 for their best leg, and 77% for their other leg, Fig 3 panels C, F, I, L, and Table 5). Strong skews (>1), significant departures from normality (Kolmogorov-Smirnov) and significant Chi-square goodness-of-fit statistics demonstrated that data from those 3 items were not normally distributed (Table 5).

**Table 5:** Skewness, test of normality and chi square results of three MABC-2 tasks in Experiment 2, including an exploratory measure calculating errors.

	MD3	B2	B3	
			Best leg	Other leg
**Exp 2**				
**Skew**				
Best	1.89 (0.27)	-1.21 (0.27)	-2.55 (0.27)	-2.55 (0.27)
Average	1.46 (0.27)	-.362 (0.27)	-1.37 (0.27)	-1.43 (0.27)
Band 3	.574 (0.27)	-.203 (0.27)	-.773 (0.27)	-.549 (0.27)
**Kolmogorov-Smirnov** Df = 81				
Best (p)	.212, (.001)	.154, (.001)	.459, (.001)	.445, (.001)
Average (p)	.180, (.001)	.148, (.001)	.235, (.001)	.253 (.001)
Band 3 (p)	.092, (.026)	.195, (.001)	.222, (.001)	.195, (.001)
**X**^**2**^ Df = (4–7)				
Best (p)	32, (.05)	135, (.001)	108, (.001)	135, (.001)
Average (p)	23, (.05)	54, (.001)	44, (.001)	66, (.001)
Band 3 (p)	5, (.57)	43, (.001)	71, (.001)	64, (.001)
**% at ceiling**	32	50	79	77
**Errors**				
**Skew** (SE)				
Best		2.110 (0.27)	3.60 (0.27)	3.38 (0.27)
Average		.878 (0.27)	2.09 (0.27)	2.38 (0.27)
Band 3		.902 (0.27)	1.16 (0.27)	1.21 (0.27)
**Kolmogorov-Smirnov** Df = 81				
Best (p)		.268, (.001)	.448, (.001)	.425, (.001)
Average (p)		.123, (.004)	.229, (.001)	.241, (.001)
Band 3 (p)		.124, (.004)	.183, (.001)	.188, (.001)
**X**^**2**^ Df = (3–9)				
Best (p)		97, (.001)	64, (.001)	54, (.001)
Average (p)		10, (.312)	26, (.001)	41, (.001)
Band 3 (p)		11, (.272)	43, (.001)	54, (.001)

Experiment 2 includes best of two trials, average across two trials and band 3 trial scores (.001) indicates p≤.001.

Similarly to Experiment 1, we performed two further tests to investigate methods that might normalize the distribution of the data. First, we took the average of both trials on the Age Band 2 items. This allowed taking into consideration that children might perform at best on the first trial but not on the second. This reduced the skewness, Kolmogorov-Smirnov, and Chi-square goodness-of-fit statistics (Fig 3 panels C, F, I, L, and Table 5), showing a reduction of ceiling effects. Second, we analyzed the scores of the Age Band 3 items. This method also reduced all the statistical test scores, showing a reduction in ceiling effects when Age Band 3 was used (Fig 3 panels C, F, I, L, and Table 5).

Next, we explored other methods of scoring the items, focusing on the time taken to perform the items, and the number of errors made. Other MABC-2 items in which performance time is measured (i.e., MD1 and MD2), although still not normally distributed, did not show as strong or as obvious ceiling effects as some untimed items. We therefore investigated whether including a time variable could improve the distributions of two of these items (B3 was not timed due to performance being too fast to time accurately by hand). It is possible that children are trading-off speed and accuracy in these items; children performing at ceiling may be taking much longer than children making some errors, but performing quickly. To investigate potential speed-accuracy trade-offs, Pearson’s (parametric) correlations were calculated and demonstrated no significant relationships between performance and time in B2 (Trial 1, *r* = .077, *p* = .498; Trial 2, *r* = .080, *p* = .478; best trial, *r* = .070, *p* = .539; mean of trials, *r* = .125, *p* = .265; Trial 3, *r* = -.106, *p* = .345). In MD3, there was also no significant linear correlation between performance time and scores in the two trials of Age Band 2 (Trial 1, *r* = -.063, *p* = .577; Trial 2, *r* = -.204, *p* = .067; best trial, *r* = -.112, *p* = .320; mean of trials, *r* = -.125, *p* = .267); but a significant negative correlation was observed on the Age Band 3 item, (Trial 3; *r* = -.323, *p* = .*003)*. Spearman’s non-parametric tests revealed the same patterns. These results suggest that time and performance are independent in these items, at least at Age Band 2. The lack of a speed-accuracy trade-off means we can therefore continue to use errors as the primary dependent variable for these items—the cause of the ceiling effects does not seem to be trade-offs in performance speed.

An alternative method for assessing performance on items B2 and B3—similar to the assessment of MD3—used the total number of errors, as opposed to just recording the first step (i.e., occurrence) on which an error was made. With this method, children who made one mistake on the first step would not get a score of 0 (lowest possible score) if they continued to perform well thereafter. This scoring method decreased the skew, Kolmogorov-Smirnov, and Chi-square goodness-of-fit statistics on MD3 and B2 for the standard (i.e., best of 2 trials) measure. When the average of two trials was used, both the B2 Age Band 2 scores and the B2 Age Band 3 scores changed from being significantly different from normally distributed (i.e., p≤.05) to being not significantly different from normally distributed (i.e., p>.05, Table 5).

Finally, as an internal check of the new measurements in this experiment, inter-rater reliability was investigated, with two out of three raters scoring each item and participant. A two-way random analysis of variance intraclass correlation coefficient (ICC) assessed the degree that coders provided consistency in their scoring across subjects. The resulting ICC values were: 0.975 for MD3, 0.946 for B2, and 0.835–0.916 for B3, indicating that all the pairs of coders had a high degree of agreement.

## A non-parametric approach

While the changes to administration and scoring of the MABC-2 performance test explored in Experiments 1 and 2 improved the distribution of the raw scores, they did not fully normalise these distributions, and ceiling effects remained. In addition, these changes in administration, if adopted widely, would require lengthy and expensive adaptation and re-norming of the MABC-2.

A better approach would be to accept that the underlying raw scores are not normally-distributed, to keep the administration of the test exactly as it is, and to embrace non-parametric statistical methods of data analysis. In the final part of our study, therefore, we investigated the usefulness of non-parametric approaches to convert raw scores on the MABC-2 to age-specific percentiles that can then be used to assess children’s motor development. This would require no adaptations to the administration and scoring of the MABC-2 test itself, would take into account the non-parametric nature of the data, and potentially make the calculations of percentiles for each item, component, and child, more accurate. A particularly useful non-parametric method involves ‘bootstrapping’—creating empirical probability distributions directly from the raw data, without any reference to standard parametric normal distributions.

Bootstrapping is a non-parametric resampling technique that estimates the underlying distribution of scores across the relevant population by repeatedly re-using the raw data taken from the sample itself. This could eliminate the problems of explicitly referring scores on each item to the normal distribution, and could in future be implemented on the original standardization dataset to adjust the tables of age-specific norms. While this would result in a completely new set of scoring tables, it would not affect the items or their instructions, therefore involving no changes to the test, while also allowing previously-acquired data to be re-interpreted in line with non-parametric statistical assumptions.

### Methods

#### Overview

The aim of the non-parametric resampling approach explored here was to use the raw data exactly as acquired under the standard procedures of the MABC-2, but with them to create new population reference distributions of scores. In principle, for each trial, item, hand or foot, component, and person, a population reference distribution can be calculated, and a percentile score can be derived that reflects each performance. Our aim here is only to match the parametric scoring procedures of the MABC-2 with a non-parametric version, and to compare the results obtained. We will report a more substantial analysis of resampling methods for the MABC-2 in the future.

#### Algorithm

The process used to construct population reference distributions for Age Band 2 items of the MABC-2 was run separately for each age group within Age Band 2 (7, 8, 9, and 10 years old). Following pilot work, a total of 300,000 iterations of the basic resampling procedure was required to cover all potential combinations of scores. Within each of these iterations, the algorithm was to:

1. Select a random child from the sample who has a full, valid data set and does not have a diagnosis of DCD
2. For each item, limb, and trial for this participant, select a random data point from all of that child’s valid data points
3. Save the best performance scores for each item, following standard MABC-2 procedures

**Fig 4:**
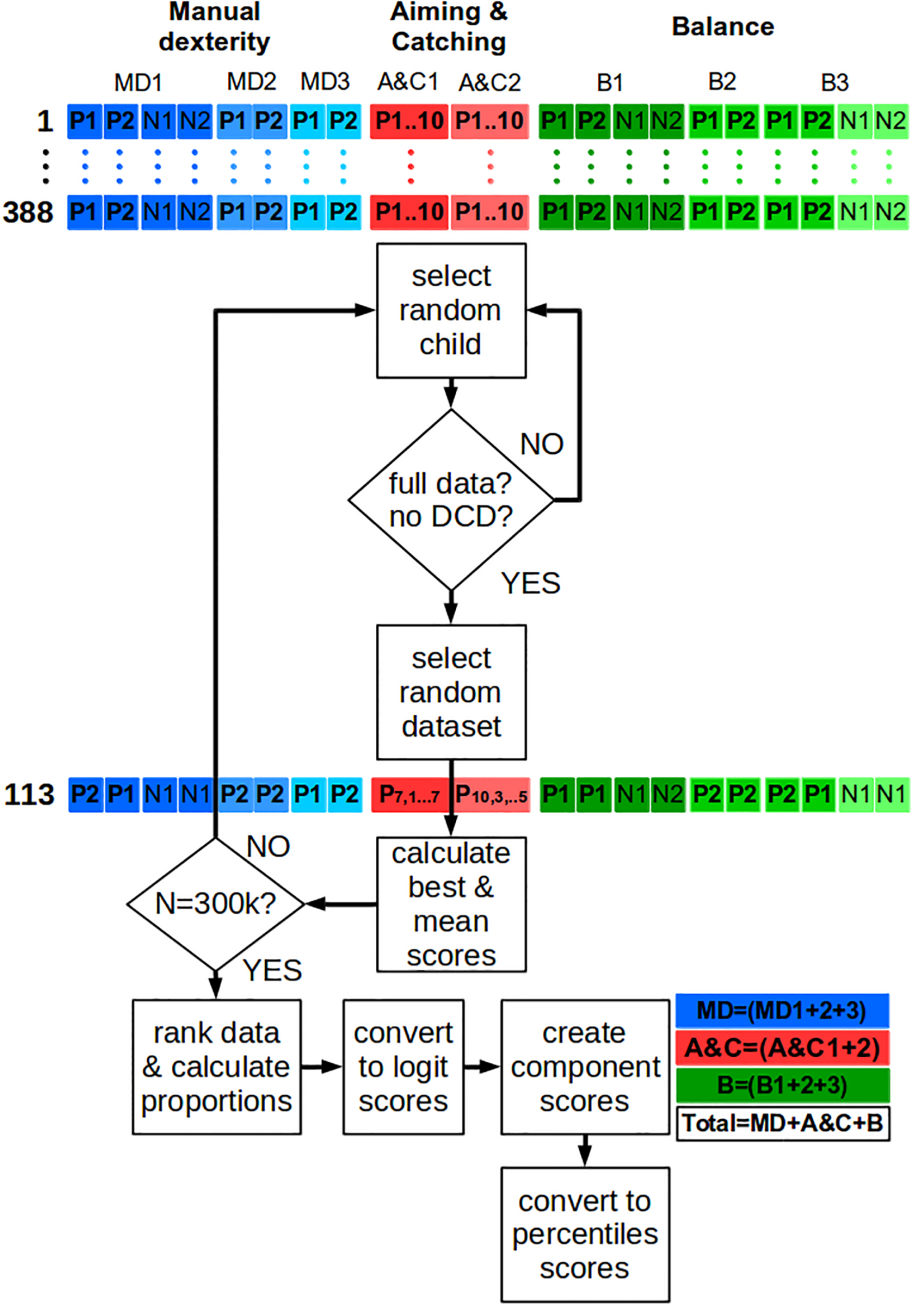
Algorithm for creating non-parametric ‘bootstrapped’ distributions of MABC-2 scores. The three components of movement (Manual dexterity, MD; Aiming & catching, A&C; Balance, B), are represented in blues, reds, and greens respectively. Each component includes 2 or 3 items, shown in different shades. Each item includes between 2 and 10 trials, executed with the dominant or preferred hand or leg (P1, P2, … P10) or the non-dominant or non-preferred hand or leg (N1, N2). The full dataset of 388 children was used. On each of 300,000 iterations of the basic resampling procedure, a random child was selected. The dataset was checked for completeness, and for the child not having a DCD diagnosis. From that child’s full dataset, a random dataset was created, by sampling, within each item and each hand or leg randomly with replacement from the 10 (A&C) or 2 (all other items) available trials. From this random dataset, the ‘best’ and ‘mean’ scores for each item, hand, and leg were calculated, and the process repeated for another randomly selected child. After 300,000 random datasets had been created, the scores were ranked, converted into proportions, and converted to logit scores. These logit scores were used to create component standard scores and a total standard score, and these standard scores were converted into percentiles.

Since items AC1 and AC2 each comprise 10 individual trials, on each of which the child could either fail (0) or succeed (1), a bootstrap sample was created in a different way. For example, a child who scored 6 out of 10 has a dataset of 4 fails and 6 successes: [0,0,0,0,1,1,1,1,1,1]. On each iteration, one trial was randomly sampled with replacement from these ten trials, then repeated for a total of ten trials. The total number of successes across these 10 resampled trials (i.e., a score from 0 to 10) was used as the bootstrap sample for each iteration. The whole procedure is illustrated in Fig 4.

#### Standard scores, component scores, total scores, and percentiles

Once all 300,000 resampled datasets had been generated for each age group, the scores for each item across 300,000 bootstrap samples were ranked from worst (0^th^ percentile) to best (100^th^ percentile). Tables were created containing the list of possible scores, and the lower and upper percentiles of datasets with these scores. Importantly, during this ranking process, scores for each resampled child remained together so that any within-child relationships between scores (i.e., across trials, items, and components), was maintained. For example, to produce a percentile for a single trial, just that trial’s scores were ranked, but to produce a percentile for a single item, the raw scores were combined across trials, and the resulting best score was ranked. For items using both preferred and non-preferred limbs, the ranking was done both across trials, and again on the average of the standardised scores (see below).

At this stage, the percentiles reflected only a single item and a single hand or foot. In order to replicate the analyses used in the MABC-2, these percentiles needed to be combined across limbs to produce item standard scores, across items to produce component scores, and across components to produce a total score. Percentiles are non-linear, non-parametric, constrained by lower (0%) and upper (100%) limits, and should not be combined without transformation. Rather than the normal distribution, to allow this combination we converted percentiles into logit scores using the logistic transformation, α = log(p/(1-p)), where α is the logit score and p is the percentile expressed as a proportion. While similar to converting data using a normal distribution, the logistic transformation is more suited to percentiles and non-parametric distributions.

Logit scores were then averaged across items which require two limbs (MD3, B1, B3), summed across components, MD = (MD1+MD2+MD3), AC = (AC1+AC2), B = (B1+B2+B3), and averaged across all items to produce a total logit score T = (MD1+MD2+MD3+AC1+AC2+B1+B2+B3)/8. Each of these scores was converted back into a percentile using the inverse logistic transformation, p = 1/(1+exp(-α)), where p is the percentile expressed as a proportion and α is the (sum of) logit score(s).

#### Re-scoring the raw data

The final step was to return to our original raw data and calculate percentiles for each trial, limb, item, component, and total score using the new, non-parametric reference tables. To compare the distributions of data under the standard parametric and the new non-parametric methods, percentiles were converted for both methods to logit scores.

### Results & discussion

#### Distributions of component and total scores

**Fig 5:**
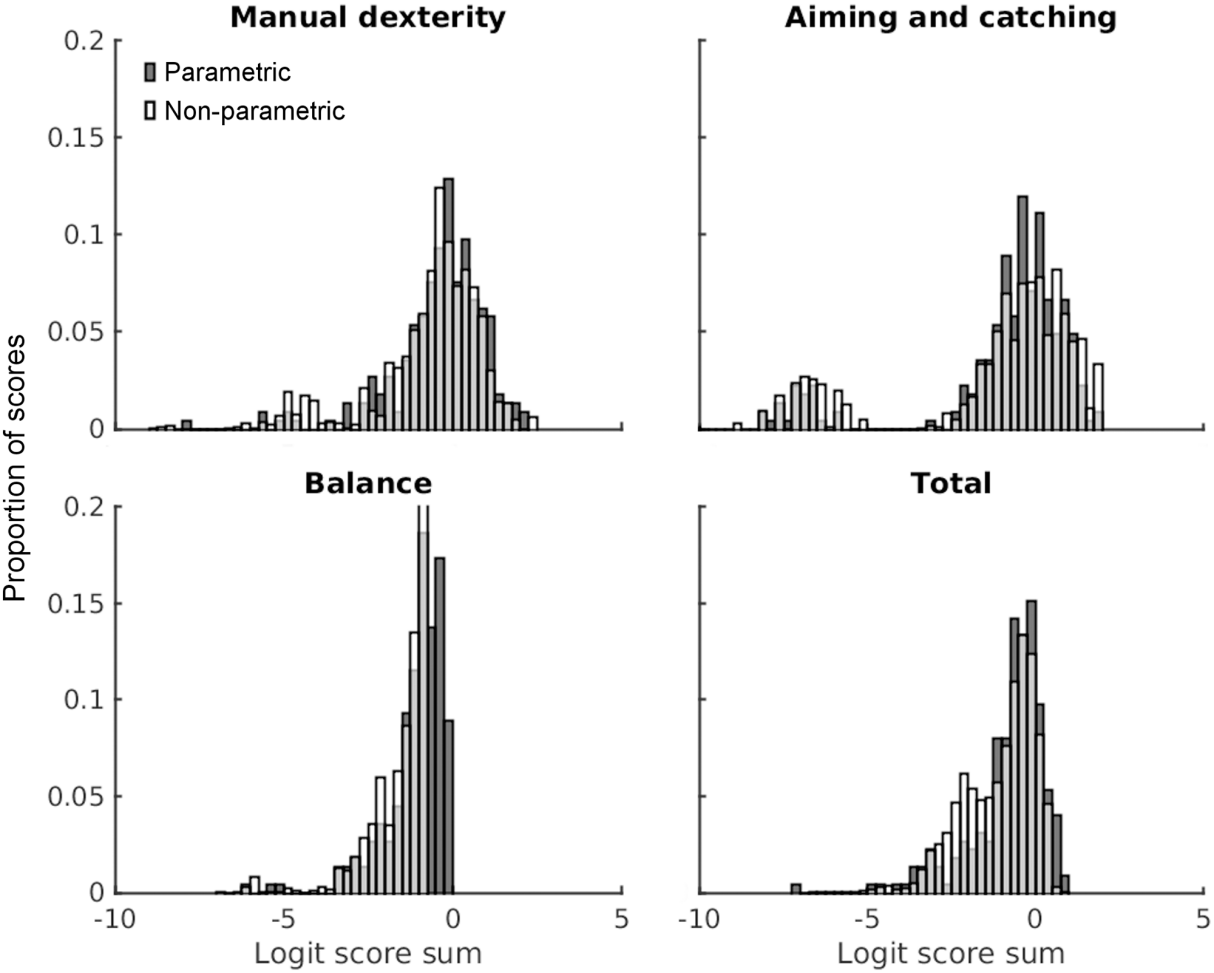
Distributions of component standard scores and total standard scores following standard parametric (filled columns) and alternative non-parametric resampling methods (unfilled columns). Component and total score percentiles were converted into logit scores and plot in bins of 0.25 width across all participants, normalising to a total plot height of 1. Note that the y-axis scale has been truncated for the non-parametric scores in the balance component. Just under 50% of all these scores were in a single bin.

Fig 5 plots the distributions of component and total scores for parametric, in dark grey filled columns, and non-parametric methods (white, unfilled columns) of scoring the MABC-2. While the distributions overlap substantially (indeed, they must, since they are derived from normalised percentile data), there are important differences, particularly in the lower and upper tails of the distributions.

First, the distributions of all scores are negatively skewed, both for the non-parametric scores (skew, MD = -1.57, AC = -1.38, B = -2.53, and T = -0.88), but also for the parametric scores (skew, MD = -1.74, AC = -1.91, B = -2.22, and T = -1.79). This is surprising, as the standardising procedure and the norms tables included with the MABC-2 should, in theory, result in unskewed, normally-distributed component and total standard scores.

Second, the mean logit scores for all distributions are also below zero. This is clearest for the non-parametric analysis of balance scores. This is due, partly, to the way in which some failures are scored in the MABC-2 –performance may be given a standard score of 1 (i.e., 3 standard deviations below the mean, regardless of the proportion of children who actually produces this result). It also seems to be due, partly, to the way in which standard scores are mapped onto normal distribution percentiles in the MABC-2 scoring system. Percentiles inferred from a standard score in the normal distribution should be interpreted as the proportion of the distribution which is *below* that score. In the MABC-2, with a mean standard score of 10, 50% of children will have standard scores *below 10*, and 50% will have scores of *10 or above*. But this does not seem to be the way in which these data are often used. While a score of 10 may be assigned to the 50^th^ percentile, the correct interpretation is that 50% of children have scores *lower than 10*; 63% of children should have scores lower than 11; so about 13% should have a score of 10 (given that scores are rounded). The overall result of using the lower-bound to assign percentiles to scores is to shift all percentiles down towards zero, and to make the mean logit score negative. Solutions to this problem will need to be dealt with in future.

Third, the most strongly-skewed raw data, from the balance items, result in the most highly-skewed component scores, and the largest differences between percentiles derived from parametric and non-parametric methods. The differences between parametric and non-parametric methods are clearly apparent for the total scores, presumably due to the combined effect of the three problems discussed above. Two-sample Kolmogorov-Smirnov tests comparing the two methods revealed that the parametric and non-parametric scores were significant differently distributed for MD (test statistic, ks = 0.115, p = .005), B (ks = 0.422, p<.001), and total scores (ks = .159, p<.001), but not for the AC scores (ks = 0.0804, p = .104). Again, since AC raw scores were approximately normally distributed, the parametric and non-parametric methods produced comparable results.

#### Relationships between standard parametric and novel non-parametric methods

**Fig 6:**
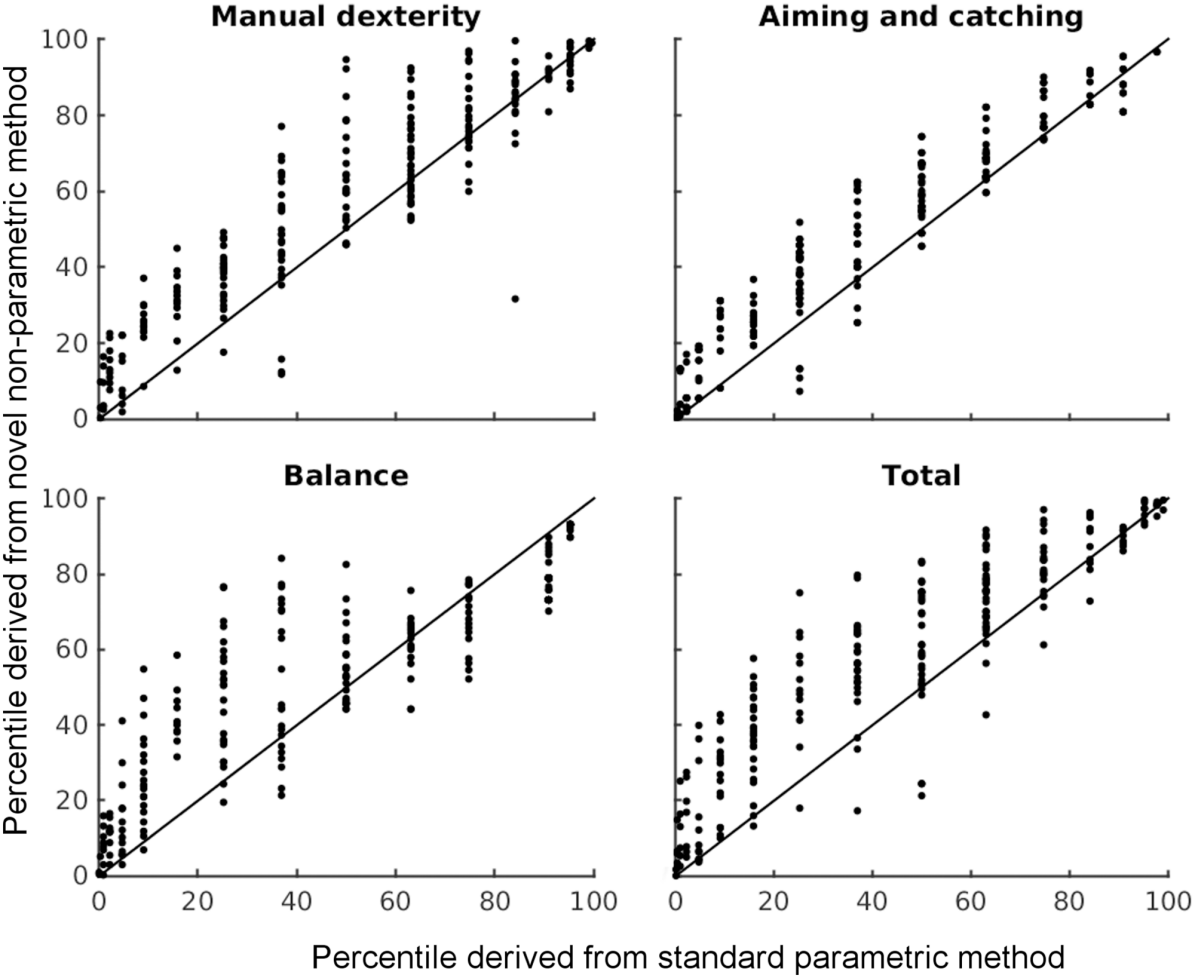
Relationship between parametric (x-axis) and non-parametric (y-axis) methods of assigning percentiles to scores for 225 8–10 year-old children performing the MABC-2. Data points above the diagonal lines indicate higher percentiles were assigned for non-parametric than parametric; below the lines indicates higher for parametric than non-parametric.

Our final analysis compared the percentile scores assigned to each child following the standard parametric and our new non-parametric approach. Non-parametric percentile scores were created by ranking the average component and total logit scores across all children in the age group. Parametric percentile scores followed the standard MABC-2 method. Fig 6 shows the relationship between parametric percentile scores on the x-axis, and our non-parametric scores on the y-axis.

First, the median non-parametric percentiles (MD = 59.5, inter-quartile range = {32.9, 80.9}%, AC = 45.8{26.2, 67.8}%, B = 56.4{31.5, 73.4}%, T = 59.2{33.6, 79.6}%) were higher than the corresponding median parametric percentiles (MD = 50{25.2, 63.1}%, AC = 25.2{4.78, 36.9}%, B = 36.9{15.9, 74.8}%, T = 36.9{15.9, 63.1}%). This reflects the problems addressed above. Second, while the rank correlations between the two measures were all significant (MD r_s_(223) = .915, AC r_s_(223) = .954, B r_s_(223) = .896, T r_s_(223) = .919, all ps<.001), across the two methods, data for the AC items were most highly-correlated, and the B items were least correlated. This again reflects the fact that aiming and catching distributions were closest to normal, and balance furthest. Finally, particularly in the middle of the performance range, there is substantial disagreement between the two methods. For example, a child given a '37^th^ percentile’ score on the balance component with the standard, parametric approach, might achieve anywhere between the 21^st^ and 84^th^ percentiles using our non-parametric approach. The differences between the percentiles arise only from how the population distributions were constructed for each age group, and how individual percentile scores were computed from those distributions—the raw data are identical.

## General discussion

The MABC-2 assesses children’s movement, and was designed specifically to identify children with motor impairments—children in the lower tail of the distribution of movement ability. The experiments and analysis presented here demonstrate the limits of some items of the MABC-2 with regards to assessing the full continuum of abilities from poor to excellent. Our data questions the validity of using the normal distribution and its parametric assumptions for describing and interpreting performance on the MABC-2.

We emphasize again that the MABC-2 is primarily designed to identify motor impairment [1], as opposed to measuring performance across the full range of motor skills. Other tests such as the BOT-2 [14] may be preferable for research purposes, and it might therefore not be necessary for the MABC-2 to be valid for assessing the full continuum of movement skills. Nonetheless, while certain MABC-2 items result in normally-distributed movement abilities (AC2, Table 1) and allow both impairment and expertise to be assessed, other items result in highly skewed distributions, demonstrate clear and strong ceiling effects, and can thus only assess impairment. This discrepancy between item distributions, however, is not reflected in the scoring scheme, with all scores converted to standardised normal scores and percentiles under the parametric assumptions of normal distributions.

For the purposes of research studies, to improve the distributions of scores on these items, and therefore to meet the parametric assumptions of the MABC-2, two potential solutions are to change the items (make them harder, or perform more movements or trials on each item), or to change the way the items are scored. We discuss each of these possibilities in turn.

### Potential modifications to the administration of the MABC-2 items

We identify four potential modifications to the MABC-2 items that may reduce the influence of ceiling effects and decrease possible violations of parametric assumptions.

1. Administer two trials on all items for all children. By administrating two trials for all children, regardless of performance on the first trial, consistency between participants can be established, as they will all perform under the same instructions and for approximately the same time, equalising fatigue and time-on-item.
2. Do not stop children when they make errors on the walking item. While the instructions of item B2 advise that children should be stopped when they make their first mistake, instructing them to carry on for all 15 steps, and counting the total number of errors, may allow for a more representative range of performance to be assessed.
3. For children who perform at ceiling on one or both of the two trials, administer a third trial on the upper Age Band. Giving the harder version as a third trial might allow for the exploration of a wider range of abilities. However, interpreting and combining children’s scores on two separate items is complicated. This complication was not addressed here.
4. Make items longer or harder (adding more trials, more steps, more hops). The items could be modified by increasing their difficulty, or the number of movements, to measure a wider range of abilities, just as in the aiming and catching items, which result in much more normally distributed scores. Low scores will still allow for impairment to be observed, while children who score highly might still struggle to perform more movements, allowing a more continuous assessment. For instance, in item B3, participants could do 10 hops instead of five, turning around at the end.

These four possibilities involving a change in item administration could improve the distribution of the data. Our first three suggestions could be implemented easily, without extensive changes in the administration of the items. While the first two may simply reflect good practise on any psychological or behavioural test, the third suggestion provides data that are difficult to interpret. Our fourth suggestion, although more demanding as it would involve a change of items, may reflect the best long-term solution to the issue of ceiling effects. All these changes would require collection of new standardised reference datasets and the increase in assessment time would also need to be taken into consideration. At present, we would not recommend making any changes to the way the items are administered or how the data are collected, especially not for clinical purposes. For the purpose of research, however, the above modifications may be useful. Instead, the most useful approach may be to embrace alternative ways of scoring the data to avoid the problems that ceiling effects introduce.

### Potential modifications to the MABC-2 measurement and scoring

We identify two possible modifications to how the MABC-2 is measured and scored that may reduce the influence of ceiling effects and decrease possible violations of parametric assumptions. First, use the average of all valid trials, not just the best score. We found that using the average across trials as opposed to the best of the two trials reduces ceiling effects in all cases. Second, use the total number of errors, not just the time of the first error. In Experiment 2, we found that, in item B2, measuring the number of mistakes reduced the distribution’s departure from normality.

These suggestions will improve the distribution of data primarily through a change in scoring, rather than test administration. The first suggestion could be implemented relatively easily, following a consistent administration of two trials for each participant. However, while this solution improved the distribution of data, it still departed significantly from being normally distributed. The use of total number of errors instead of the instance of the first error was shown to greatly improve the distribution of data from item B2, and could be implemented with minimum change in item administration.

Our final suggestion, to use non-parametric methods, would eliminate the problems of using the normal distribution and its parametric assumptions, and could be implemented retrospectively on the original standardization dataset to create new scoring tables. While this would mean a complete change of scoring tables, it would not affect the items, their instructions, or the way the raw data are initially processed (i.e., using the best, rather than the mean performance). Adopting non-parametric norms would involve minimal changes, and allow previously-acquired data to be re-interpreted in line with the new tables.

### Bootstrapping to produce new norms for the MABC-2

Our bootstrapping analyses revealed substantial differences in the distributions of scores derived from parametric and non-parametric methods, particularly for the balance items. The reasons for these differences need to be explored further, but candidates include the way the raw data are processed, the way the standardisation dataset was transformed to the normal distribution, the low difficulty of the items, and the simple fact that the raw data from MABC-2 items are not normally distributed. Attempts to transform these discrete, non-normal distributions with strong ceiling effects into normal distributions are unlikely to succeed.

Further, it was not simply the case that the parametric and non-parametric distributions were differently, but still smoothly and consistently transformed by the processing stages. Rather, the rank-ordering of participants’ component and total scores changed substantially. On some components, knowing the parametrically-derived percentile score (i.e., the ranking), particularly in the middle of the distribution, predicted relatively poorly the likely ranking from the non-parametric scores. This was most severe for the balance, and least severe for the aiming and catching data—since aiming and catching data were mostly normal, the parametric and non-parametric methods produced comparable results. These discrepancies arise, we believe, because the component and total standard scores are derived by pooling across items with both normally-distributed, and non-normally distributed scores. Without knowing the full details about how the original MABC-2 dataset were standardised, it is difficult to isolate the reasons for the reported discrepancies.

### Other considerations

Our conclusions and suggestions are necessarily limited to certain items of the Age Band 2 of the MABC-2. The distributions of other Age Band items still need to be assessed to generalize our findings. Further, we do not yet know which of the two methods—parametric or non-parametric—is better. Despite all that we have discussed, if the parametrically-derived scores make better diagnostic predictions, or better predict performance on other movement assessments than the non-parametric scores, then the standard scoring system should remain in place. It is beyond the current scope to assess the reliability or validity of the potential new scoring system that we have explored here.

Similar issues to the ones we have discussed were found in the MABC-2 Checklist (1) with observable floor effects as children get older. While the original authors emphasized that the MABC-2 does not intend to measure a continuum of abilities but rather focuses on impairments, the prior observation of floor effects led to the use of non-parametric statistical procedures for further analyses of the MABC-2 Checklist. Until new non-parametric standardization tables are available for the MABC-2 performance test, we suggest that the use of raw scores, rather than item standard scores, component standard scores, total standard scores, or percentiles may be preferable for research-related analysis. Using the MABC-2 percentiles is also potentially problematic given the relatively small numbers of children tested in each year group, both in the original standardisation dataset [1] and the present work (i.e., N = 51–145 per year group). Relatively small differences in performance might therefore have quite large effects on the percentile scores assigned; sample-sizes in the thousands are likely to be required for a much finer-grained assessment of movement (e.g., [15–16]).

One important final consideration, raised by our reviewers, is that the MABC-2 performance test implicitly assumes that there are three distinct underlying (i.e., latent) domains of movement ability (manual dexterity, aiming & catching, balance) along with one overall, or general movement ability [17–18]. In this manuscript, we have focused primarily on the raw data and the analysis of movement across the three domains of movement performance, the 8 test items, and 21 individual trials across both hands and legs. Whether and how these individual test items should be combined into one or more domain-specific or general movement ability scores is beyond the scope of the present report, but represents a critically-important challenge for the MABC-2 that is also relevant for other movement assessments such as the BOT. Meeting this challenge will likely require further development of statistical methods and models using, for example, item response theory, the use of Rasch analysis, and ‘person-independent’ calibration of performance test items [15,17–18]. Whichever statistical approach is used to measure children’s movement ability, the theoretical models will be constrained or influenced by the quality and distribution of the raw performance data they are based upon.

## Conclusion

We found that at least three of the eight MABC-2 items show clear ceiling effects and produce data that are not normally distributed. While task modifications lessen the influence of these effects, non-parametric methods may be required to interpret MABC-2 scores reliably. The suggestions made in our study may be of primary importance for research purposes, and for the strict use of quantitative data under parametric assumptions. In clinical settings, the quantitative and qualitative data gathered together by the MABC-2 allow for guidance on the observation of impairment and diagnosis. These qualitative observations on large numbers of children have highlighted many areas of ambiguity that cannot easily be scored quantitatively, such as differing quality of movement, or lack of attention, reinforcing the need for qualitative observation. The MABC-2, while useful in clinical settings and assessment of individuals, might not be as efficient for quantitative research and larger numbers of participants. Future versions of the test may need to take into consideration the measurement and scoring issues, and the potential solutions that our study has raised.
