# Correction: Ceiling effects in the Movement Assessment Battery for Children-2 (MABC-2) suggest that non-parametric scoring methods are required

**DOI:** 10.1371/journal.pone.0202689

**Published:** 2021-01-20

**Authors:** Blandine French, Nicole J. Sycamore, Hannah L. McGlashan, Caroline C. V. Blanchard, Nicholas P. Holmes

There are errors in the Funding section. The correct funding information is: This research was supported by the Medical Research Council, UK (grant number MR/K014250/1 to NPH). The funders had no role in study design, data collection and analysis, decision to publish, or preparation of the manuscript.
